# Prevalence and predictive factors of testosterone-induced erythrocytosis: a retrospective single center study

**DOI:** 10.3389/fendo.2024.1496906

**Published:** 2025-01-15

**Authors:** Anina Neidhart, Viktor von Wyl, Benno Käslin, Christoph Henzen, Stefan Fischli

**Affiliations:** ^1^ Department of Internal Medicine, Division of Endocrinology, Diabetes and Clinical Nutrition, Luzerner Kantonsspital, Lucerne, Switzerland; ^2^ Institute for Implementation Science in Healthcare, University of Zurich, Zurich, Switzerland; ^3^ Epidemiology, Biostatistics and Prevention Institute, University of Zurich, Zurich, Switzerland; ^4^ Control & Simulation Department, Faculty of Aerospace Engineering, Delft University of Technology, Delft, Netherlands

**Keywords:** hypogonadism, testosterone replacement therapy, erythrocytosis, predictive factors, risk factors

## Abstract

**Aim:**

This study analyzes the prevalence and predictive factors of testosterone-induced erythrocytosis (TIE) in patients receiving testosterone replacement therapy (TRT).

**Methods:**

Retrospective single-center observational study.

**Results:**

247 patients were included; median age was 47.0 years (interquartile range (IQR) 32-60) and median follow-up years 2.9 (1.0-5.5). The most common indication for TRT was central hypogonadism (51%) followed by primary hypogonadism (26%). TRT was carried out with testosterone undecanoate (TU) n=194, testosterone enanthate (TE) n=18 and testosterone gel (n=35). Compared to baseline, hematocrit (HCT) values at last follow-up (LFU) increased significantly by +0.04 (95% confidence interval (CI) [0.027, 0.050], p=<0.0001) in all patients (n=92) and +0.06 (95%CI [0.031, 0.057], p<0.0001) in the TU group (n=71). 57% of the patients reached an HCT value>0.46, 23% >0.5 and 5%>0.54. 46% of the patients who have reached an HCT value >0.46 have had their highest HCT measurement within the first year of TRT application. Logistic regression analysis indicated that body mass index (BMI) was significantly associated with the development of an HCT ≥0.5 (p=0.013) and HCT ≥0.46 (p=0.008). There was an association between the baseline HCT measurement and the outcome of a HCT measurement ≥0.46 (p=0.025), patients with high starting values were more likely to develop TIE.

**Conclusions:**

TIE appears to be frequent and does not only present within the first year of therapy which indicates a close follow-up of laboratory values within the first year followed by annual controls. Baseline BMI and baseline HCT measurement should be considered in risk stratification of TIE development.

## 1 Introduction

Since decades testosterone replacement therapy (TRT) represents a standard procedure for treatment of primary and secondary hypogonadism and in persons with gender dysphoria (female-to-male) (1). Therefore, different testosterone formulations are applied transdermal (i.e., testosterone gel), intramuscular as depot injections (testosterone enanthate, testosterone undecanoate) and peroral as capsules (testosterone undecanoate). The latter form of application is more and more abandoned due to restricted availability and a unreliable bioavailability (2, 3).

In general, TRT represents a safe therapy but regular clinical and biochemical control of persons receiving TRT is mandatory. One of the most common side effects is development of secondary erythrocytosis following initiation of TRT or Testosterone-Induced Erythrocytosis (TIE) (4–7).

Erythrocytosis is defined as an erythrocyte count above the normal and sex-specific range, this refers to a hemoglobin level and hematocrit respectively of ≥16.5 g/dL/0.49 in men and ≥16.0 g/dL/0.48 in women (8) but different definitions exist. In contrast to primary erythrocytosis where – in most cases – a myeloproliferative neoplasm (i.e., polycythemia vera [PV]) induces a overproduction of red blood cells, polyglobulia in secondary erythrocytosis is the result of either a hypoxic stimulus (i.e., tissue hypoxemia in chronic obstructive pulmonary disease and smoking), paraneoplastic erythropoietin overproduction (i.e., in renal cell carcinoma) or is medicament-induced as in TIE.

Testosterone metabolism and erythropoiesis are closely linked. There is an association between hematocrit (HCT)-/and hemoglobin-levels and the concentrations of total and free testosterone (9). Testosterone injections exhibit a dose-dependent effect on the production of red blood cells in healthy men and this effect seems to be more pronounced in older subjects (10, 11).

Several pathophysiological mechanisms leading to TIE are discussed: Testosterone is aromatized to estradiol which could have a direct stimulatory effect on hematopoietic stem cell proliferation (12). However, clinical data demonstrate that this is not an essential prerequisite for the induction of erythrocytosis (13). Testosterone reduces hepcidin, an important regulator of iron metabolism, resulting in an increased resorption and bioavailability of iron and consecutively stimulation of erythropoiesis (14, 15). The exact role of hepcidin downregulation under physiologic and pathologic conditions has still to be defined as – at least in animal models – hepcidin seems not to have a crucial function in the regulation of testosterone-mediated erythropoiesis (16). Other studies describe the impact of alternative endocrine regulators as dihydrotestosterone on TIE (17).

On a clinical basis, additional risk situations as co-factors for erythrocytosis in patients undergoing TRT always must be considered. Apart from smoking, the presence of an untreated obstructive sleeping apnea syndrome (OSAS) increases the risk of TIE (18) and newer medications like the inhibitors of the sodium-glucose cotransporter 2 (SGLT-2i) can induce or aggravate erythrocytosis (19–22).

The clinical consequences of erythrocytosis can be severe, the increase in blood cell volume are associated with higher blood viscosity and therefore a possible higher risk for thromboembolic events (23), especially in patients with PV (24, 25).

There is conflicting data if TIE confers a higher risk for venous thromboembolism (VTE) and/or major adverse cardiovascular events (MACE, i.e., myocardial infarction, stroke or cardiovascular death) in testosterone recipients (26–29). However, current guidelines suggest dose adaptation or stopping of TRT when erythrocytosis develops (1, 30–32).

The prevalence of erythrocytosis in the available literature is varying considerably. In general persons treated with testosterone exhibit a three- to four-fold risk of erythrocytosis (33, 34). In the literature and depending on the definition of erythrocytosis and the mode of application prevalence rates between 7 and 66.7% are reported (35, 36). This is also true for persons receiving gender-affirming hormonal therapy, where a prevalence between 11 and 20% is described (5, 37).

The formulation of the testosterone product (i.m. vs. p.o. vs. transdermal) seems to play a role in regard to the risk for erythrocytosis whereas short-acting testosterone esters (testosterone enantate) bearing the highest risk and transdermal the lowest (4–6, 38, 39). However, less is known about risk factors, the time course of development of TIE and the absolute risk predisposing patients with TRT to develop TIE. In this single center and retrospective study, we studied over a follow-up period of several years the prevalence and development of TIE and assessed risk-factors for development of erythrocytosis under TRT.

## 2 Materials and methods

### 2.1 Patient selection

**Figure 1:**
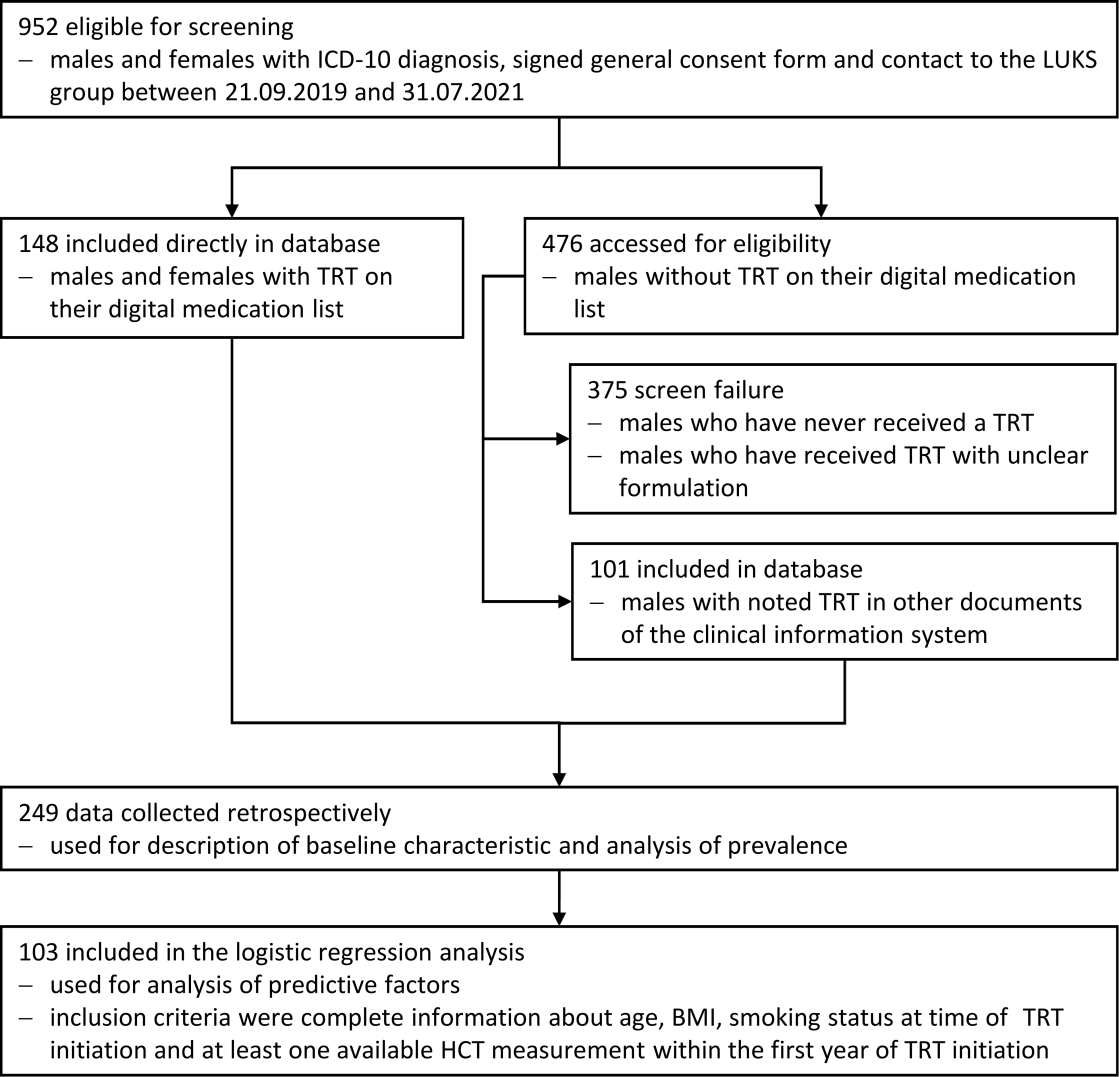
Flow chart indicating patient selection.

This retrospective study investigates patients who have developed erythrocytosis after the beginning of TRT at the Luzerner Kantonsspital (LUKS) since 2013. The clinical information system was searched for patients with specific ICD-codes (international classification of diseases) representing indications for TRT and the database was retrospectively compiled using the information that could be found in the electronic health records. We could identify 952 persons eligible for screening finally 247 were included in the data analysis and 103 in the logistic regression analysis (**Figure 1**). We included only patients with organic causes of central or primary hypogonadism. Persons with functional hypogonadism (suppression of the hypothalamus-pituitary-testis axis due to comorbidities, i.e., diabetes mellitus or obesity) were excluded.

Testosterone was dosed in most cases according to the label (250mg testosterone enanthate every 3-4 weeks i.m.; 1000mg testosterone undecanoate every 10-14 weeks i.m.; testosterone gel 40-50mg applied on the skin every morning). Control of testosterone dose (testosterone gel) or injection interval (intramuscular formulations) was monitored by determination of a total testosterone level 2-4h after gel-application or a trough testosterone level immediately before the next injection (after having achieved steady-state conditions). Biochemically adequate substitution was defined by total testosterone levels in the mid-normal reference range (gel) or at the lower end of the reference range (intramuscular formulations). The overwhelming use of TU relies on the fact that this medication is the only preparation in Switzerland that is reimbursed by the insurance.

Laboratory values were collected at baseline (before starting the TRT) and approximately 3, 6 and 12 months after TRT and at last follow-up (most recent value) respectively. In addition, we registered every HCT-value ≥0.46. In a 2-step approach, all patients that had an HCT measurement of >0.46 were screened for their highest-ever measured HCT value since TRT initiation until last follow-up.

### 2.2 Laboratory methods

Total testosterone (reference range: 6.68-25.7 nmol/L) and sex hormone-binding globulin (SHBG)- (20.6-76.7 nmol/L) values were measured using and electrochemiluminescence immunoassay (ECLIA, cobas e^®^ system, Roche Diagnostics, Mannheim, Germany). Hemoglobin (127-163 g/L) values were determined by photometry (sodium lauryl sulphate (SLS) hemoglobin method, Sysmex, Kobe, Japan) and hematocrit (0.37-0.46) by impedance measurement (Sysmex, Kobe, Japan).

### 2.3 Statistical methods

Data were analyzed descriptively by calculating percentages for categorical and medians [interquartile ranges] for continuous variables. Changes in HCT variables were described a means (standard deviations) at different time points (3 months, 6, months, 12 months), stratified by baseline HCT group defined by HCT tertiles.

We also investigated associations of reaching different HCT thresholds (>0.46, > 0.50, >0.54) during the first follow-up year or during the full follow-up phase with pre-specified variables. These variables included age, smoking at baseline, body mass index, presence of Obstructive Sleep Apnea Syndrome, and use of SGLT2 Inhibitors, which were analyzed using multivariable logistic regression mode (n=103) that included all variables, as well as the total follow-up duration transformed into restricted cubic splines (to adjust for unequal follow-up time of individual patients). For the subgroup of patients with a baseline HCT available (n=80), the model was re-estimated using the same variables and baseline HCT in addition.

The database was checked with an algorithm for simple human errors which occurred during the data collection. The algorithm checked if the dates of the laboratory values were in chronological order e.g., it checked if every patient’s measurement at 3 months had an earlier date than the same patient’s measurement at 6 months. Furthermore, the algorithm checked if single values had impossible values e.g., a hematocrit value >1.00.

No missing data were replaced.

### 2.4 Ethics

The study was performed in accordance with the Declaration of Helsinki and has been approved by the corresponding ethics committee «Ethikkommision Nordwest- und Zentralschweiz (EKNZ)» (Project-ID: 2021-01371).

## 3 Results

A total of 247 patients were included in the study, median age was 47.0 years (interquartile range (IQR) 32-60) and median follow-up years 2.9 (1.0-5.5). The most common indication for TRT was central hypogonadism (n=127, 51%) mostly due to a pituitary adenoma followed by primary hypogonadism (n=65, 26%). In total 15 persons (6%) with gender dysphoria were included. All patients had testosterone values in the hypogonadal range at baseline (4.94 nmol/L, [1.69-7.59]). TRT was carried out with i.m.-injection of testosterone-esters (testosterone undecanoate [TU] n=194, testosterone enanthate [TE] n=18) and testosterone gel (n=35). Other baseline characteristics are displayed in **Table 1**.

**Table 1:** Baseline characteristics.

		Testosterone undecanoate	Testosterone enanthate	Topical Gel	Total
		(n=194)	(n=18)	(n=35)	(n=247)
**Age (years)**		47.0 [33–59]	24.0 [18.2-45.2]	56.0 [38.2-68]	47.0 [32–60]
**BMI (kg/m^2^)**		28.1 [24.7-31.4]	25.1 [22.3-25.9]	29.2 [26.1-31.9]	28.0 [24.5-31.4]
	BMI 18.5 to <25 kg/m^2^	48 (25%)	10 (56%)	7 (20%)	65 (26%)
	BMI 25.0 to <30 kg/m^2^	73 (38%)	5 (28%)	15 (43%)	93 (38%)
	BMI >30.0 kg/m^2^	63 (32%)	2 (11%)	12 (34%)	77 (31%)
	Unknown BMI	10 (5%)	1 (6%)	1 (3%)	12 (5%)
Etiology					
	Central hypogonadism	103 (53%)	5 (28%)	19 (54%)	127 (51%)
	Primary hypogonadism	53 (27%)	1 (6%)	11 (31%)	65 (26%)
	Klinefelter syndrome	22 (11%)	1 (6%)	2 (6%)	25 (10%)
	Gender identity disorder	7 (4%)	6 (33%)	2 (6%)	15 (6%)
	Other	9 (5%)	5 (28%)	1 (3%)	15 (6%)
Smoking Status					
	Present or past smoker	61 (31%)	7 (39%)	13 (37%)	81 (33%)
	Nonsmoker	104 (54%)	8 (44%)	18 (51%)	130 (53%)
	Unknown smoking history	29 (15%)	3 (17%)	4 (11%)	36 (15%)
Chronic Diseases					
	Hyperlipidemia	37 (19%)	4 (22%)	4 (11%)	45 (18%)
	Diabetes mellitus	38 (20%)	2 (11%)	4 (11%)	44 (18%)
	Sleep apnea	35 (18%)	2 (11%)	6 (17%)	43 (17%)
	Chronic kidney disease	25 (13%)	3 (17%)	6 (17%)	34 (14%)
	Thyroid disorders	22 (11%)	1 (6%)	3 (9%)	26 (11%)
	Asthma	9 (5%)	1 (6%)	2 (6%)	12 (5%)
	Chronic liver disease	6 (3%)	0 (0%)	2 (6%)	8 (3%)
Additional Medications					
	Thyroid hormones	63 (32%)	8 (44%)	12 (34%)	83 (34%)
	Glucocorticoids	62 (32%)	6 (33%)	13 (37%)	81 (33%)
	DDAVP	14 (7%)	2 (11%)	1 (3%)	17 (7%)
	SGLT2-inhibitors	10 (5%)	0 (0%)	0 (0%)	10 (4%)
	GH substitution	5 (3%)	0 (0%)	1 (3%)	6 (2%)
	Diuretics	19 (10%)	0 (0%)	6 (17%)	25 (10%)
Laboratory Parameters					
	Hemoglobin (g/L)	140 [129–147]	142 [135–148]	142 [137–152]	141 [130–148]
	Hematocrit	0.41 [0.39-0.43]	0.43 [0.39-0.44]	0.43 [0.41-0.44]	0.41[0.39-0.43]
	Testosterone (nmol/L)	5.08 [1.64-7.60]	3.68 [1.78-5.65]	4.54 [2.14-6.03]	4.94 [1.69-7.59]

For categorical variables: n (%), for continuous variables: median [IQR].

**Figure 2:**
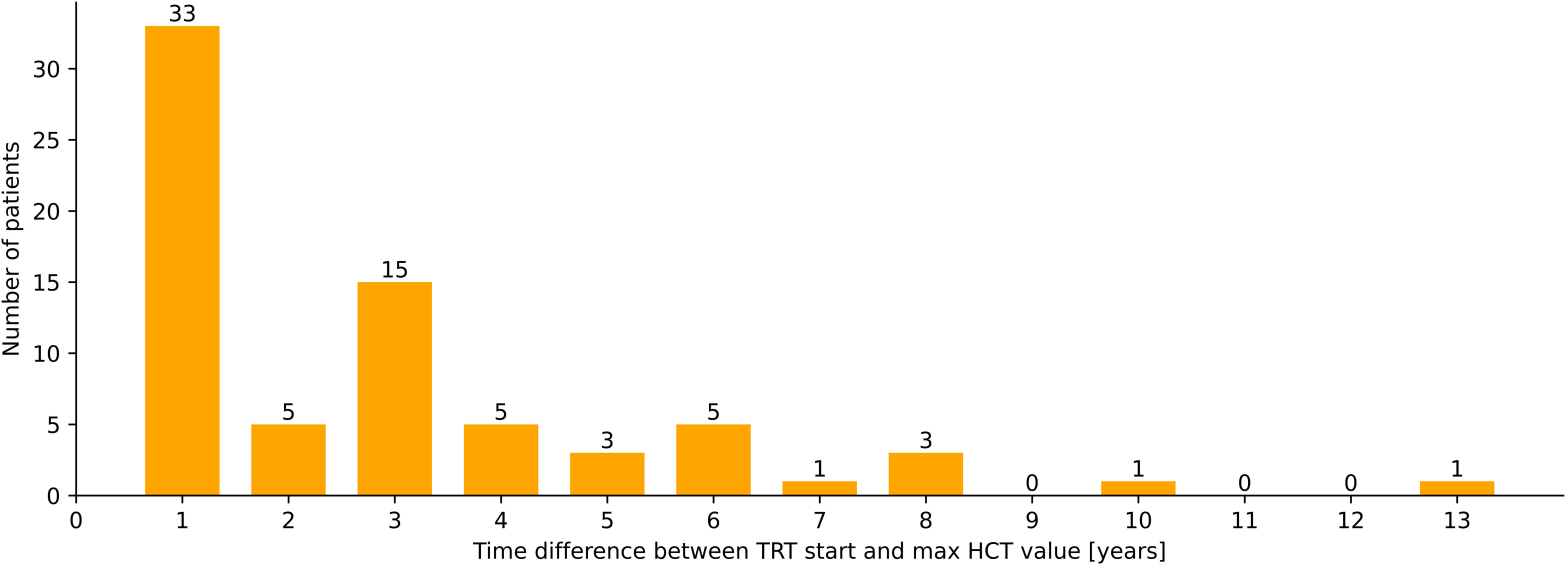
Latency between start of TRT and maximal HCT-values (years), n=72.

A total of 94 patients had baseline HCT values. Compared to baseline, HCT-values at last follow-up (LFU) increased significantly by +0.04 (95% confidence interval (CI) [0.027, 0.050], p=<0.0001) in all patients (n=92), +0.06 (95%CI [0.031, 0.057], p<0.0001) in the TU-group (n=71), +0.015 (95%CI [-0.036, 0.066], p=0.48) in the TE (n=6) and +0.02 (95%CI [-0.012, 0.056], p=0.18) in the gel-group (n=15) (**Figure 2**), respectively. A total 57% (n=142) of the patients in the cohort reached an HCT-value >0.46, 23% (n=56)>0.5 and (n=12) 5%>0.54. The form of TRT application (TU vs. TE vs. gel) and basal testosterone values (before start of TRT) were not associated with a statistically significant effect on the HCT values in our sample (data not shown).

**Figure 3:**
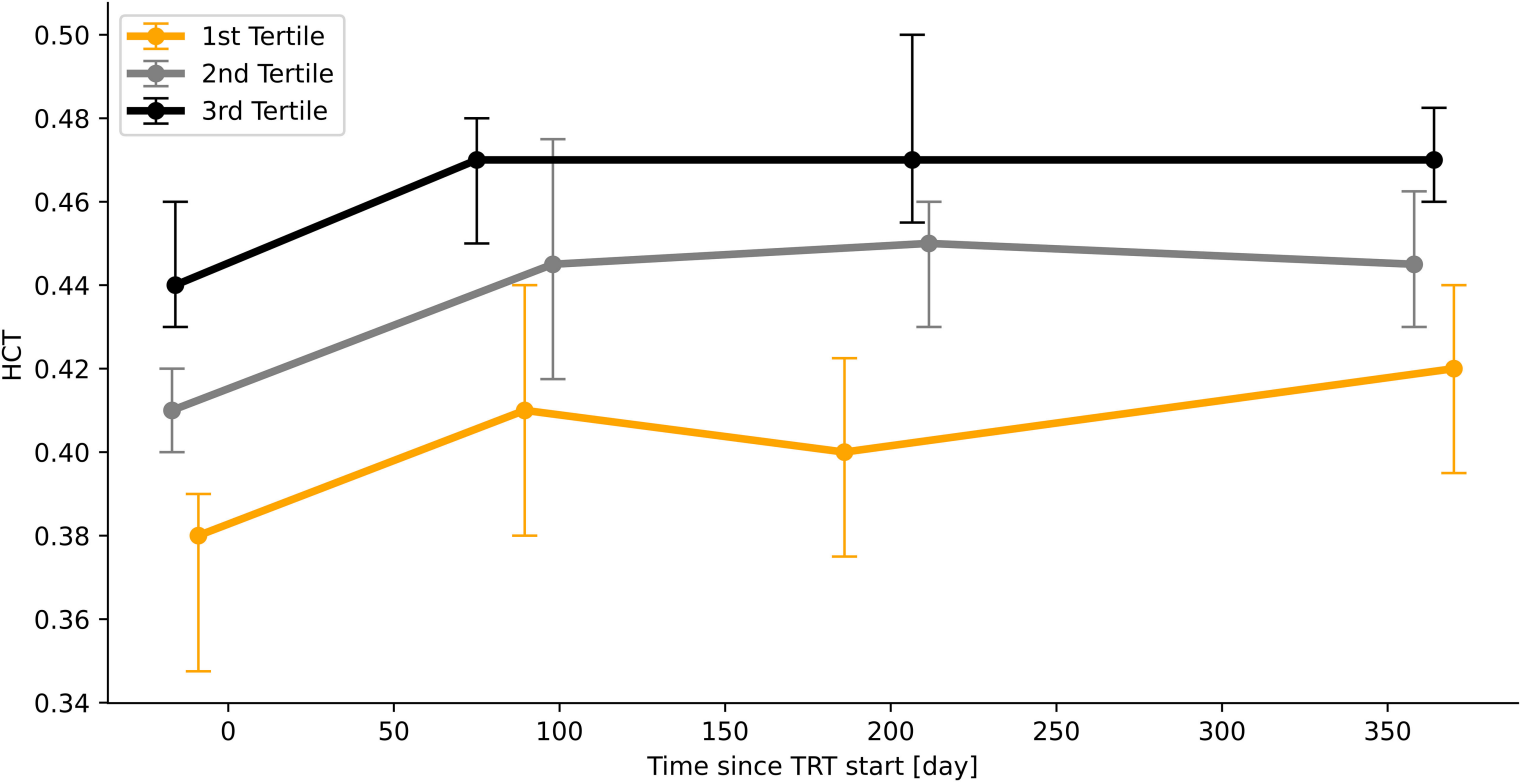
Time course of HCT-values according to initial HCT-value (stratified into tertiles). Median values, bars indicate IQR.

To describe the time course of HCT measurements as a function of the baseline HCT value, patients were stratified into tertiles (**Figure 3**, n=94). This approach suggests that the patients who have a high baseline HCT value will remain to have high values and patients who have a low baseline value overall remain at a low HCT value.

**Figure 4:**
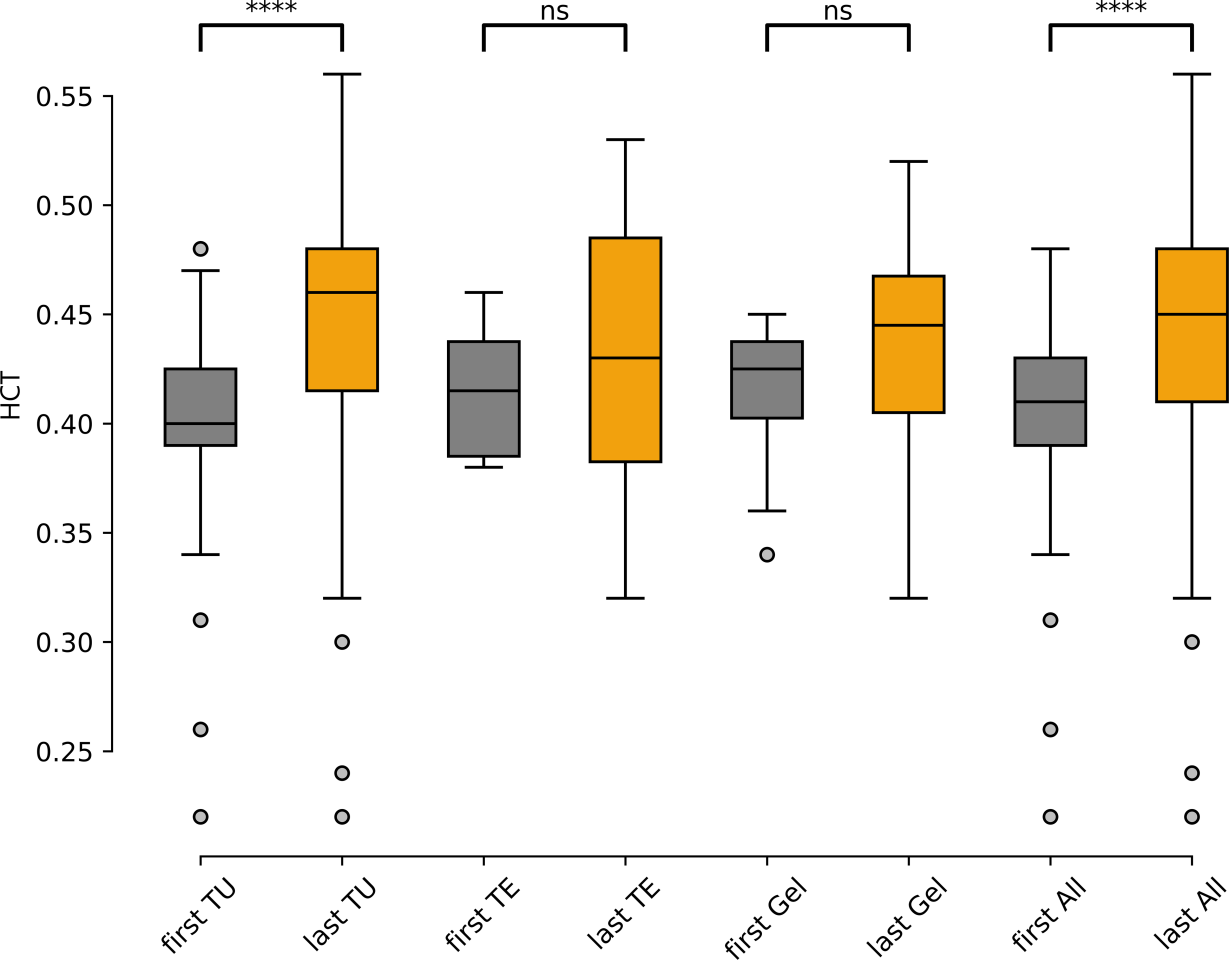
Hematocrit values before TRT («first») and at last-follow-up («last»), separated by application modality (TU, TE, Gel) and in all patients («All»), (n=92). TU, testosterone undecanoate (n=71), TE, testosterone enanthate (n=6), Gel, testosterone gel (n=15). ns: not significant; ****: p<0.0001.

The time difference between the first application of testosterone and the appearance of supraphysiological or pathological HCT values varies greatly. Not all patients reach their highest HCT values within the first year of therapy. Only 46% (n=33) of the patients who have reached an HCT value >0.46 have had their highest HCT measurement within the first year of TRT application in the other 54% (n=39) TIE appeared later (**Figure 4**).

Logistic regression (**Table 2**) analysis indicated that body mass index (BMI) was significantly associated with development of a HCT ≥0.5 (p=0.013, 1.13 [1.03; 1.25]) and HCT ≥0.46 (p=0.008, 1.15 [1.04; 1.28]). Furthermore, there was an association between the baseline HCT measurement and outcome of a HCT measurement ≥0.46 (p=0.025), which supports the observations that were in the descriptive approach. There is a trend that smoking at baseline might have an association with having a HCT measurement of ≥0.5 (p=0.086, 2.92 [0.86; 9.92]). Logistic regression analysis could not show an association between elevated HCT and presence of OSAS and the use of SGLT-2-inhibtors.

**Table 2:** Multivariable logistic regression analysis.

Full sample (n=103)	Threshold HCT ≥ 0.46	p-value	Threshold HCT ≥ 0.5	p-value
	n=59 odds ratio [95% CI]		n=37 odds ratio [95% CI]	
Active smoking at baseline	1.81 [0.47; 6.98]	0.390	2.92 [0.86; 9.92]	0.086
Age, per year increase	1.00 [0.98; 1.03]	0.918	1.00 [0.97; 1.03]	0.954
Body Mass Index, per unit increase	1.15 [1.04; 1.28]	0.008	1.13 [1.03; 1.25]	0.013
Obstructive Sleep Apnea Syndrome	1.21 [0.34; 4.36]	0.769	2.83 [0.57; 14.13]	0.205
SGLT2 Inhibitors	0.64 [0.09; 4.31]	0.646	2.56 [0.22; 29.48]	0.451
Sub-sample with baseline HCT available (n=80)				
Baseline haematocrit greater than median (0.41)	4.71 [1.21; 18.29]	0.025	2.70 [0.75; 9.66]	0.127

## 4 Discussion

Our findings suggest that TIE is very frequent in patients with testosterone substitution therapy. Over half of all patients who received TRT reached a supraphysiological HCT value of ≥0.46, 23% and 5% reached an HCT-value of ≥0.5 and ≥0.54 respectively, the latter representing a threshold where halting TRT is indicated. Interestingly the majority (54%) of the patients developed their peak HCT values after the first year of treatment indicating that regular control of red blood cell parameters is warranted. The most common testosterone formulation in our cohort was TU. Application of TU resulted in a significant median increase in the HCT value by 0.06. This is higher than previously reported (39). We also describe a higher prevalence of TIE using TU than in other cohorts (36). The reasons for this remain unclear but it can be hypothesized that different indications, follow-up intervals and patient characteristics could explain these differences.

Risk stratification, i.e., identification of patients that are prone to develop TIE in the clinical setting is crucial. However, there is paucity of data exploring risks for TIE in patients undergoing TRT. Facing the multifactorial pathogenesis of testosterone-induced erythrocytosis, different risk factors and cumulative risk when several parameters coincide must be considered. Baseline HCT seems to be predictive for the course of hematocrit during therapy. Patients with a baseline value of 0.41 or more tended to have high measurements in the follow-up and this could also be shown in the logistic regression analysis. Smoking is a known cause of polyglobulia caused by an increased red cell mass and/or reduction of plasma volume (and therefore often classified as «relative» polyglobulia) (40). A trend indicating the impact of smoking on the HCT value was shown in our data analysis. SGLT-2-inihibitors are a newer class of medication, now commonly used in the treatment of diabetes mellitus type 2, heart failure and kidney disease. Severe forms of reversible erythrocytosis in SGLT-2-users without and with concomitant TRT have been described, mainly because of hemoconcentration due to diuresis and direct effects on iron- and/or hepcidin metabolism (19–22). In our cohort only 10 patients were treated with SGLT-2-inhibitors. However, the use of these medicaments was not significantly associated with higher HCT-values in the logistic regression analysis, probably explained by the small sample size. Coviello et al. demonstrated more pronounced effects of testosterone enanthate injections on hemoglobin/HCT in persons aged 60 years or older (11). In our study population age was not a predictive factor for the development of erythrocytosis, which could be attributed to the younger mean age of our patients (47 years).

There is still controversy if OSAS is a relevant cause of secondary erythrocytosis. Whereas some studies indicate a slight increase of hematocrit in patients with OSAS, severe or clinically relevant erythrocytosis is very rare and mainly associated with severe untreated OSAS and profound hypoxemia (41–45). Lundy et al. (18) found an association of untreated OSAS and erythrocytosis in men with TRT. However, our analysis did not confirm this finding, potentially because most of our patients had treated OSAS or only light forms without relevant hypoxemia. In addition, our cohort may have included people with undiagnosed OSAS, as no general screening with polysomnography was performed in all patients.

We found a statistically significant association of having a TIE and the baseline body mass index in our logistic regression analysis. With increasing body weight, the chance of developing an erythrocytosis with HCT-values ≥0.46 and ≥0.5 respectively was increased. In the literature there is evidence that BMI and visceral fat mass are directly associated with hemoglobin and hematocrit i.e., that higher BMI increases red blood cell mass (46–48). This could be explained by a) increased aromatization of testosterone to estradiol in the fat tissue and consecutively stimulation of erythropoiesis (12) and/or b) insulin-related stimulation of red blood cell synthesis as Barbieri et al. showed that hyperinsulinemia and insulin resistance are directly related to erythropoiesis (49).

The results of this study emphasize the importance of individually adapted testosterone replacement therapy for the patient, as already pointed out in guidelines (32). This includes risk stratification and, in the presence of corresponding risk factors such as elevated baseline hematocrit or obesity, a dosing strategy aimed primarily at preventing TIE.

The strength of this study is the inclusion of a broad spectrum of patients with different indications for TRT representing a real-life scenario. In addition, patients with functional hypogonadism were excluded in this analysis. This form of hypogonadism is caused by diseases (i.e., obesity) that are associated per se with an increased risk of erythrocytosis.

However, this study has limitations. We included patients with variable treatment regimens/application forms and different co-medications. Despite stemming from a large patient population of a central hospital, the final sample size of our study was limited. In particular, group sizes of TE. and gel-treated patients was small and impacts the generalizability of our results.

The retrospective nature resulted in missing data and the timepoints of blood sampling (time between last testosterone application and determination of HCT) were variable in some cases. However, the exact timepoint between HCT-measurement and testosterone application is of importance mainly in patients treated with short-life preparations (TE). In our study this patient group was small (n=18) and therefore we believe that validity of the results has not been affected. Data regarding the smoking status (i.e., persons who quitted smoking) during the study period were not assessed and ultimately changes of application mode (i.e., switching from TU injection to transdermal forms) in the same patient during follow-up both could have had an impact on the studied parameters. We did not perform polysomnography in all patients in this study. Therefore, we cannot exclude a potential consequence of undiagnosed OSAS on TIE in our patient sample. Finally, differences in mechanisms of TIE-induction and clinical characteristics between trans- and cis-males could have an impact on the outcomes. However, the sample size of persons receiving gender-affirming hormonal treatment was small and applied testosterone doses were comparable between the two groups.

In conclusion TRT is often associated with clinically significant erythrocytosis which can appear delayed in time. Apart from baseline HCT-values and smoking status, BMI showed the strongest association with the development of erythrocytosis. Due to the multifactorial pathophysiology, different risk factors and cumulative risk must be considered in the assessment and identification of patients at risk. Long-term follow-up and regular determination of HCT in patients treated with TRT is critical.

## Data Availability

The raw data supporting the conclusions of this article will be made available by the authors, without undue reservation.
